# A Rare Case of Benign Osteoblastoma of the Mandible

**DOI:** 10.7759/cureus.25799

**Published:** 2022-06-09

**Authors:** Sneha Krishnan, Vinod K Krishna, Senthilnathan Periasamy, Santhosh P Kumar, Murugesan Krishnan

**Affiliations:** 1 Oral and Maxillofacial Surgery, Saveetha Dental College and Hospital, Chennai, IND

**Keywords:** benign tumors, recurrence risk, osteoblastoma, bone tumors, mandible

## Abstract

Benign osteoblastoma is an uncommon, solitary, osteoid, bone-producing tumor containing a rich vascularized delicate fibrous stroma and active osteoblasts. Benign osteoblastoma is a unique benign bone neoplasm that mostly affects the vertebrae and long tubular bones and rarely affects the maxillofacial skeleton. Many bone-producing lesions have clinical, radiological, and histological features that are similar to osteoblastoma. Benign osteoblastoma manifests clinically as localized swelling of the jaw, presenting as an asymptomatic or a symptomatic lesion, and proper investigations are necessary for accurate diagnosis. It usually manifests in the second and third decades of life predominantly in males. In this report, we present a case of benign osteoblastoma of the mandible in a 39-year-old male patient. The lesion was treated by complete surgical excision, and uneventful wound healing was observed during the one-year postoperative follow-up period.

## Introduction

Osteoblastoma presents as a benign, slow-growing neoplasm of the bone that is characterized by the proliferation of numerous osteoblasts that generate bony trabeculae and osteoid in a well-vascularized fibrous connective tissue stroma. It accounts for about 1% of all primary bone tumors [1]. Benign osteoblastoma is a unique benign bone neoplasm that mostly affects the vertebrae and long tubular bones. It is a rare entity in the maxillofacial skeleton accounting for 15% of the cases, and the most common location is the mandible [2]. The frequency of osteoblastoma in the mandible is high compared to the maxilla, and it is commonly found in the proximity of the tooth root. Very few cases of benign osteoblastoma of the mandible have been reported in the literature [3]. In this report, we present a case of benign osteoblastoma of the mandible in an adult male patient.

## Case presentation

**Figure 1:**
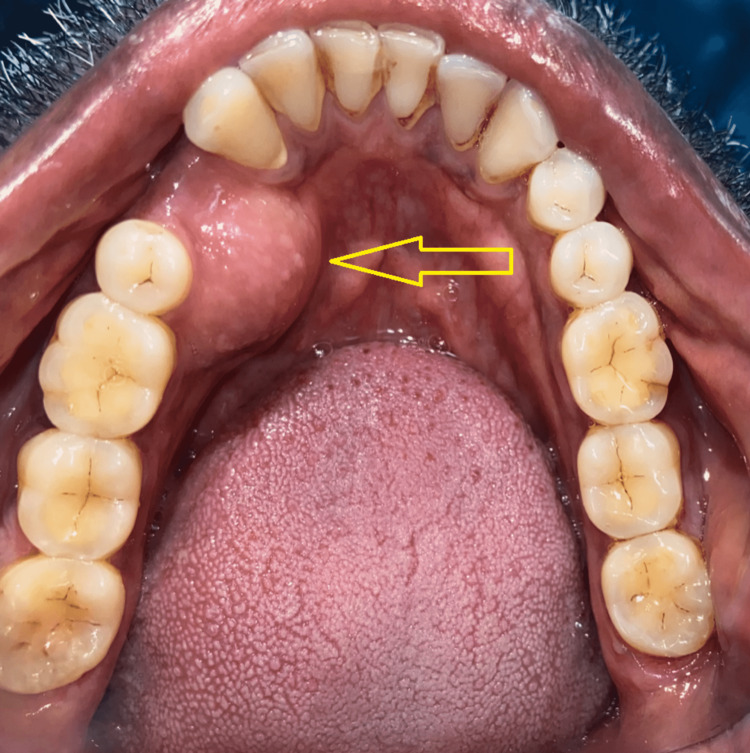
Intraoral view in mirror image showing the lesion in the right mandibular alveolar region (arrow).

A 39-year-old male patient reported to the Department of Oral and Maxillofacial Surgery with a chief complaint of growth in the right lower jaw region for the past eight years. History revealed that the growth was initially smaller in size and gradually increased to the present size following the extraction of 44. There was no significant systemic or family history. The swelling was associated with mild intermittent pain. On intraoral examination, a single localized growth measuring around 2 cm × 5 cm in its largest dimension was seen in the lingual aspect of the right posterior mandibular region. The growth extended anteroposteriorly on the medial aspect from the 43 to 46 region (Figure 1). The overlying mucosa was intact, non-erythematous, with well-defined boundaries. On palpation, the growth was non-tender, firm, hard in consistency with a lobulated surface and well-defined borders, and attached to the underlying bone. The surrounding structures were normal, and the lesion did not yield to pressure, with its base being sessile and noncompressible. Aspiration of the lesion yielded negative results, mouth opening was adequate, and regional cervical lymph nodes were not palpable. The patient’s liver function test and thyroid function test values were within normal limits.

**Figure 2:**
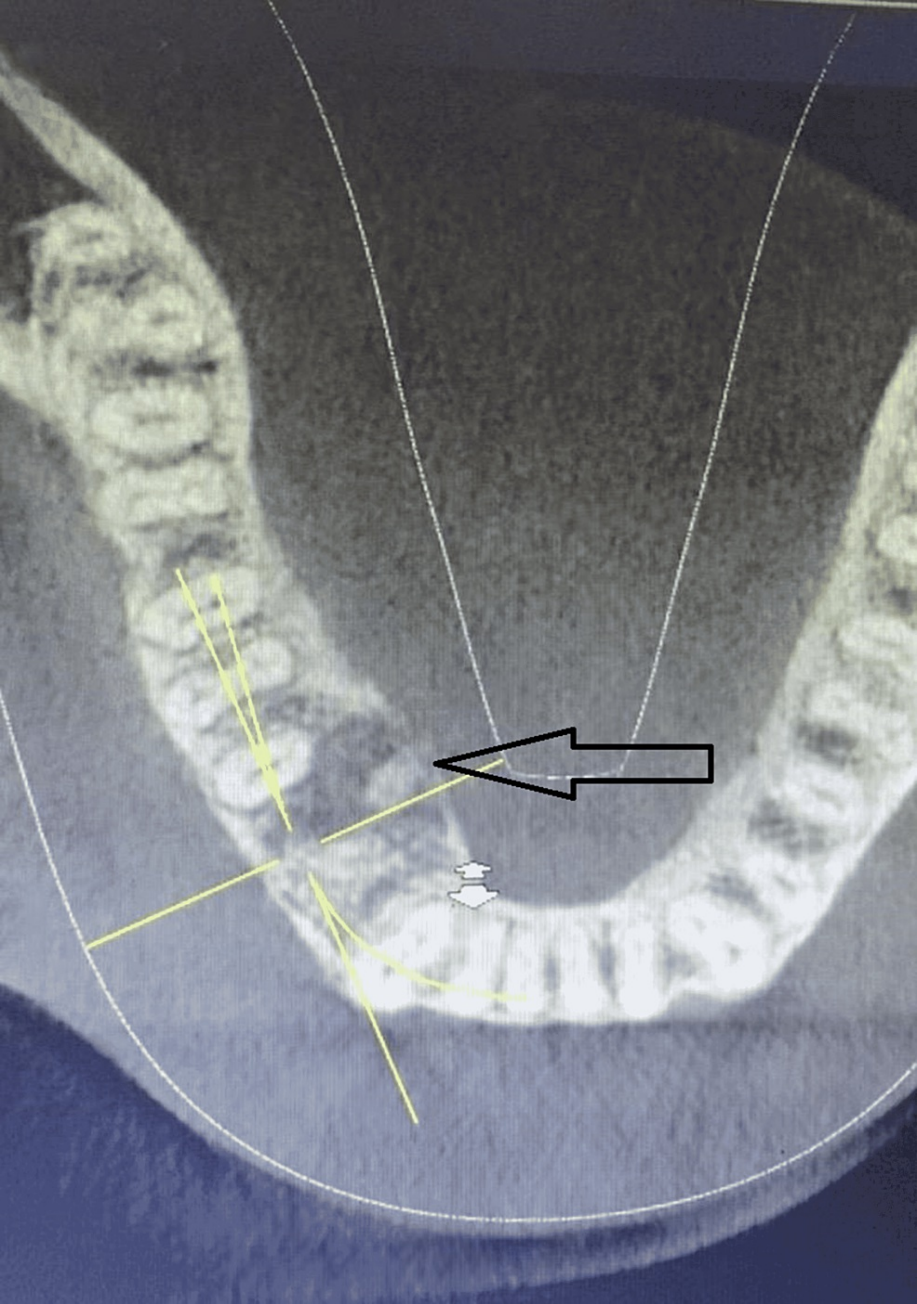
Cone-beam computed tomography scan showing radiopaque nonhomogeneous mass with calcification flecks on the lingual aspect of the right bicuspid region (arrow).

The lesion was clinically diagnosed as a benign bone tumor based on the history and clinical presentation. Differential diagnosis of the lesion consists of benign osteoblastoma, brown’s tumor, ossifying fibroma, osteoid osteoma, and fibrous dysplasia. Cone-beam computed tomography scan of the lower arch revealed a radiopaque nonhomogeneous mass with calcification flecks that were oriented toward the lingual surface in the right bicuspid region with a compact trabecular pattern (Figure 2). There were no signs of tooth displacement and root resorption.

**Figure 3:**
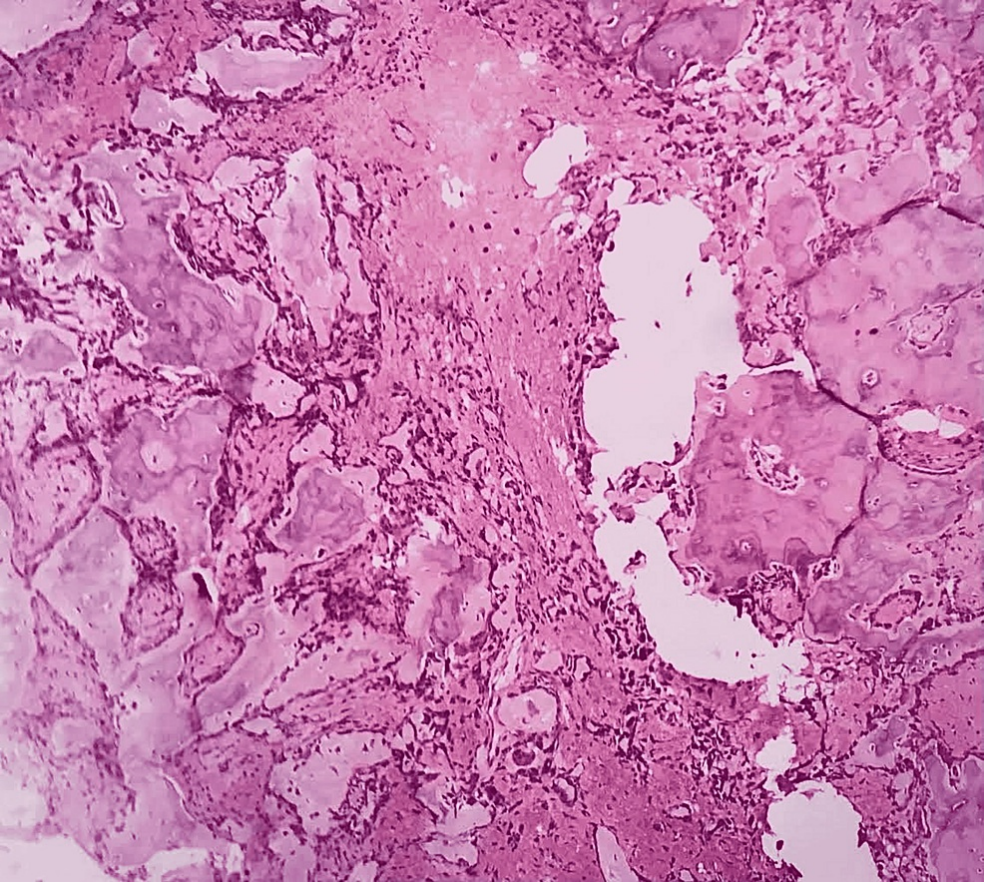
Histological examination shows features typical of a benign osteoblastoma (hematoxylin and eosin stain, 100× magnification).

An incisional biopsy was performed under local anesthesia. Histopathology revealed irregular, broad trabecular with irregular reversal lines suggestive of osteoid trabeculae exhibiting osteoblastic rimming in many areas and osteoclasts evident within the resorptive lacunae. Numerous giant multinucleated cells were also found in the fibro-cellular connective tissue stroma along with an intense chronic inflammatory cell infiltrate which predominantly consisted of lymphocytes, intense vascularity, and areas of hemorrhage (Figure 3). Thus, the histopathological finding was suggestive of benign osteoblastoma.

**Figure 4:**
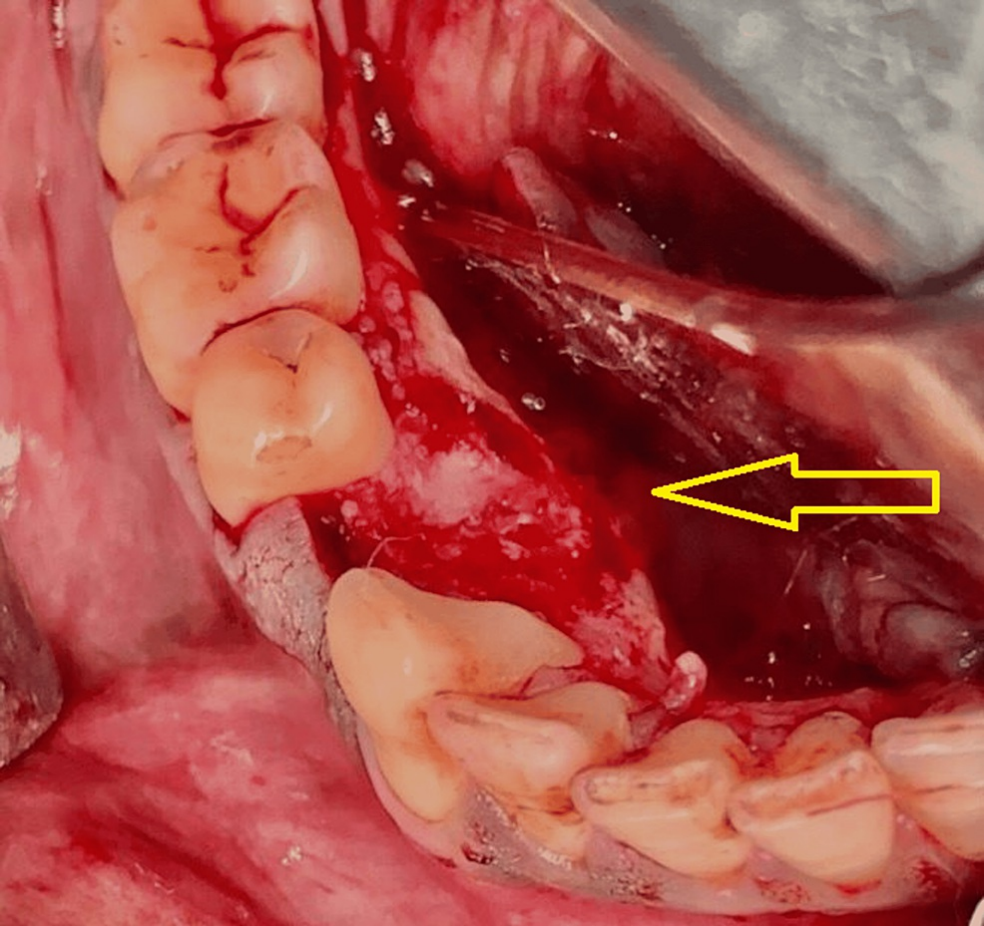
Surgical exposure of the lesion in the lingual aspect of the right posterior mandibular region (arrow).

**Figure 5:**
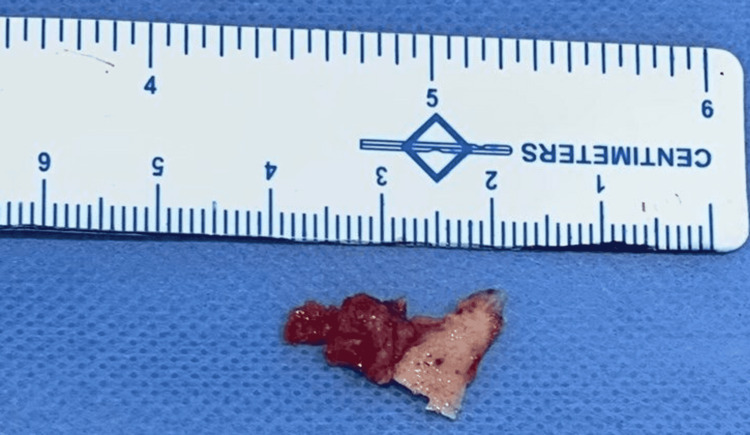
Osteoblastic lesion excised in toto.

Based on history, clinical and radiological features, blood investigations, and histopathological findings, the lesion was confirmed as benign osteoblastoma and was planned for surgical excision under general anesthesia. A crevicular incision was placed from the 41 to 47 region, and a full-thickness mucoperiosteal flap was elevated. The bone tumor was identified and exposed (Figure 4). Punch cuts were given with surgical bur, and the osteoblastic lesion was surgically excised in toto (Figure 5).

**Figure 6:**
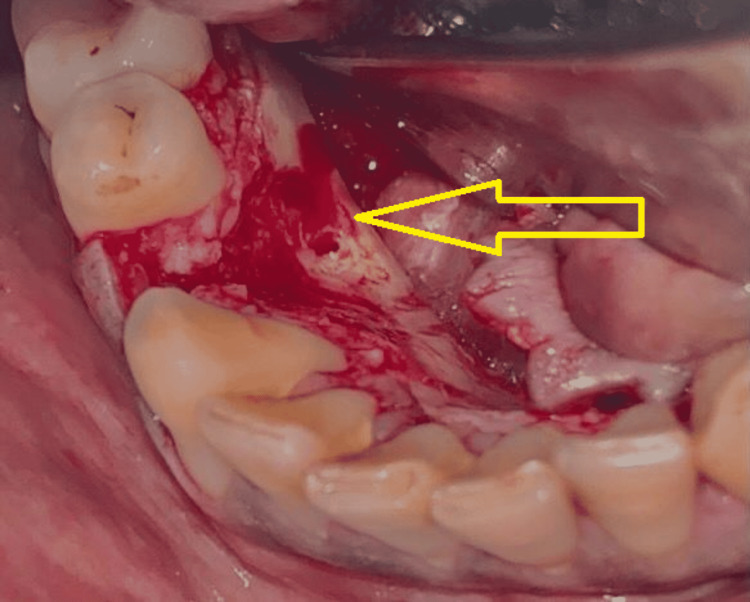
Surgical site after excision of the lesion (arrow).

**Figure 7:**
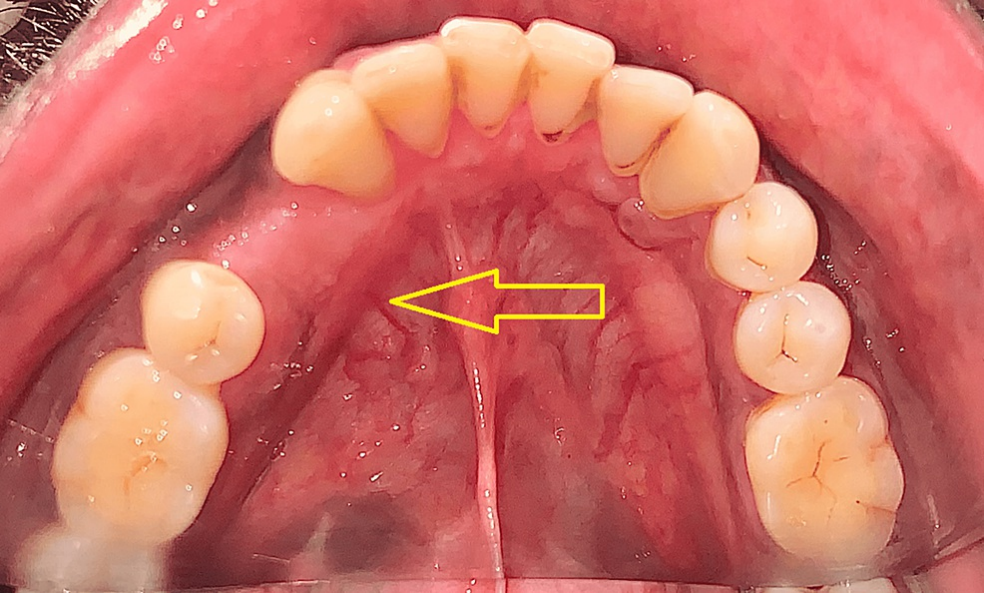
Intraoral view in mirror image showing good wound healing during the one-year postoperative follow-up period (arrow).

Bony recontouring of the lesion was done which was followed by peripheral ostectomy (Figure 6). Hemostasis was obtained and wound closure was done. During the one-year postoperative follow-up period, the wound healing was satisfactory and presented with no signs and symptoms of recurrence (Figure 7).

## Discussion

Osteoblastoma is a benign solitary bone neoplasm that accounts for about 1% of all bone tumors and 3.5% of benign bone tumors [1]. It is described as an osteoblastic osteoid tissue-producing neoplasm containing an abundance of osteoblasts. The exact nature of osteoblastoma remains unknown, despite it being a benign tumor of osteoblastic origin. It is thought to arise as an aberrant local reaction of the injury to tissues due to inflammation or perhaps a localized modification in the physiology of bone [4]. Conventional osteoblastoma is a biologically benign tumor that seldom grows larger than 4 cm in size and has restricted growth potential. A subset of borderline osteoblastoma is locally aggressive with growth exceeding 4 cm in size and is difficult to classify as “standard” osteoblastoma or osteosarcoma. They have been divided into two categories, namely, osteoblastoma-like osteosarcoma and malignant osteoblastoma or aggressive osteoblastoma [5].

Benign osteoblastoma, an intramedullary lesion, can also arise subperiosteally. Long bones, vertebral column, innominate bone, talus, patella, sacrum, calvarium, rib, scapula, metacarpals, and metatarsals are the most common sites for osteoblastoma [6]. It rarely involves the posterior region of the mandible and maxilla. It usually manifests in the second and third decades of life predominantly in males with a male-to-female ratio of 2:1 [7]. Our case exhibited a rare presentation in the mandible posterior region in an adult male patient.

Diagnosis of benign osteoblastoma is difficult due to its uniqueness, perplexing clinical-radiologic presentation, and histopathologic characteristics that occasionally mimic osteosarcoma and other bone tumors. Cementoblastoma, osteoid osteoma, and benign osteoblastoma have histologic similarities, thus implying a relationship between these pathologic conditions [8]. Owing to the benign nature and location of this tumor, curettage or conservative surgical excision remains the treatment modality, and en bloc resection is seldom performed. A simple and thorough curettage results in complete wound healing in most cases with no recurrence [9].

Radiotherapy should be considered if surgical excision is not possible and there is evidence of persistent aggressive behavior of the tumor or recurrence. Improperly excised tumors have been reported to have a 14% local recurrence rate [10,11]. The majority of the osteoblastoma is treated by curettage or surgical excision with the placement of bone grafts if required. Benign osteoblastoma has a good prognosis after surgery. Long-term monitoring is recommended due to the risk of recurrence and to evaluate the survival and integration of the grafts [12,13].

## Conclusions

Benign osteoblastoma is an uncommon bone neoplasm that affects the maxillofacial region. Very few cases of benign osteoblastoma of the mandible have been reported in the literature. This case report adds information to the nature of this pathology and highlights the significance of adequate surgical excision for successful outcomes. Several bone tumors resemble osteoblastoma clinically, radiologically, and histologically, and adequate knowledge is essential in diagnosing and managing this rare entity. Though osteoblastoma of the maxillofacial skeleton is a very rare entity, it should be considered in the differential diagnosis of bony swellings of the jaws. Benign osteoblastoma is best treated by curettage or conservative surgical excision. Proper surgical resection eliminates the risk of recurrence and results in a good prognosis.
